# Age‐Stratified Associations of Sarcopenic Obesity With Mortality in Type 2 Diabetes

**DOI:** 10.1002/jcsm.70211

**Published:** 2026-01-28

**Authors:** Shinta Yamamoto, Yoshitaka Hashimoto, Fuyuko Takahashi, Moe Murai, Nozomi Yoshioka, Yuto Saijyo, Chihiro Munekawa, Hanako Nakajima, Noriyuki Kitagawa, Takafumi Osaka, Ryosuke Sakai, Hiroshi Okada, Naoko Nakanishi, Saori Majima, Emi Ushigome, Masahide Hamaguchi, Michiaki Fukui

**Affiliations:** ^1^ Department of Endocrinology and Metabolism Kyoto Prefectural University of Medicine, Graduate School of Medical Science Kyoto Japan; ^2^ Department of Diabetes and Endocrinology Matsushita Memorial Hospital Moriguchi Japan; ^3^ Department of Diabetes and Metabolism Osaka Railway Hospital Osaka Japan; ^4^ Department of Diabetology Kameoka Municipal Hospital Kameoka Japan; ^5^ Department of Endocrinology and Diabetology Ayabe City Hospital Ayabe Japan; ^6^ Department of Fundamental Science Kyoto Institute of Technology Kyoto Japan

**Keywords:** complication, mortality, sarcopenic obesity, Type 2 diabetes mellitus

## Abstract

**Background:**

Sarcopenic obesity, the coexistence of sarcopenia and obesity, has been linked to adverse health outcomes due to comorbidity. Evidence on the association between sarcopenic obesity and mortality among individuals with Type 2 diabetes remains limited

**Methods:**

Sarcopenic obesity was defined using Japan Working Group on Sarcopenic Obesity criteria. Participants were divided into four groups based on the presence of sarcopenia and obesity. Cox proportional hazards models evaluated the mortality risk. Subgroup analyses were performed by age (40–75 vs. >75 years). Sensitivity analysis was performed by dichotomizing participants into sarcopenic obesity and non‐sarcopenic obesity groups.

**Results:**

Of the 799 participants (mean age 68.6 years, 59.3% men), proportions of neither sarcopenia nor obesity, obesity alone, sarcopenia alone and sarcopenic obesity were 56.2%, 34.5%, 6.3% and 3.0%, respectively. During a median follow‐up of 46 months, 41 deaths occurred. Compared with neither of them, the adjusted hazard ratios (aHRs) (95% CI) of mortality in obesity alone, sarcopenia alone and sarcopenic obesity were 0.53 (0.18–1.57) (*p* = 0.25), 2.36 (0.99–5.6) (*p* = 0.053) and 2.89 (1.01–8.30) (*p* = 0.048), respectively. Age‐stratified analyses revealed that sarcopenic obesity markedly increased mortality risk in participants aged 40–75 years (aHR 13.1 [2.93–58.4], *p* < 0.001), whereas sarcopenia alone (aHR 3.21 [1.07–8.33], *p* = 0.004), but not sarcopenic obesity (aHR 1.41 [0.34–8.81], *p* = 0.51), was associated with increased mortality in those aged > 75 years. Compared with non‐sarcopenic obesity, sarcopenic obesity had a significantly higher hazard ratio for mortality (aHR 4.0 [1.44–11.0], *p* = 0.008). In age‐stratified analysis, this association remained significant in participants aged 40–75 years (aHR 14.1 [3.25–61.5], *p* < 0.001), but not in those aged > 75 years (aHR 1.55 [0.32–7.46], *p =* 0.59) (interaction *p* = 0.03).

**Conclusions:**

In Japanese individuals with Type 2 diabetes, sarcopenic obesity was significantly associated with increased mortality risk. This relationship was particularly pronounced in individuals aged 40–75 years, whereas sarcopenia alone was associated with increased mortality risk rather than sarcopenic obesity in individuals aged > 75.

## 1 Introduction

With the progression of an ageing society, a decline in physical function has become a significant factor affecting healthy life expectancy. In particular, sarcopenia that is an age‐related loss of skeletal muscle mass and strength has been closely associated with increased risks of mortality and cardiovascular disease (CVD) [1]. Sarcopenia is also frequently observed in individuals with Type 2 diabetes, and its impact on health outcomes has attracted growing attention [2, 3].

In recent years, the concept of sarcopenic obesity, defined as the coexistence of sarcopenia and obesity, has been proposed. In 2022, international diagnostic criteria were published jointly by the European Society for Clinical Nutrition and Metabolism (ESPEN) and the European Association for the Study of Obesity (EASO) [4]. Similar to sarcopenia alone, sarcopenic obesity has been reported to be associated with increased risks of physical disability, cardiovascular and metabolic diseases [5, 6, 7] and a higher mortality risk [8, 9] and has emerged as a clinically important condition.

It has been reported that, compared to non‐diabetic individuals, individuals with diabetes in particular Type 2 diabetes tend to have higher body fat content despite having similar body mass index (BMI) values [10]. This suggests that sarcopenic obesity is more likely to occur in individuals with diabetes [11].

Previous studies have shown that sarcopenic obesity in individuals with Type 2 diabetes is associated with macroalbuminuria and macrovascular complications and may increase the risk of chronic kidney disease (CKD) progression [12] and mortality [13]. However, studies demonstrating the relationship between sarcopenic obesity and mortality in individuals with Type 2 diabetes remain limited, with most focusing exclusively on older individuals. As such, the current body of evidence is insufficient.

Although the diagnostic criteria proposed by ESPEN and EASO are widely used, they may not adequately reflect the physical characteristics of Asian populations. Compared to Europeans, Asians tend to have lower skeletal muscle mass, and even mild obesity may have a greater impact on insulin resistance and the progression of atherosclerosis [14]. Given these racial and cultural differences, the ESPEN–EASO criteria have limitations in accurately evaluating sarcopenic obesity in Asian populations. Accordingly, there has been a growing demand in Asian countries, including Japan, for diagnostic criteria that consider racial and cultural characteristics.

In response to this need, the Japan Working Group on Sarcopenic Obesity (JWGSO) has developed new diagnostic criteria for sarcopenic obesity that are applicable to Asian populations [15]. Although sarcopenia is generally considered an age‐related disease, it is not confined to older adults. The JWGSO criteria are applicable from middle age onwards. In addition, the JWGSO criteria were derived using large‐scale Japanese cohort data and expert consensus that linked muscle mass, muscle strength and body fat percentage with functional impairment and adverse health outcomes in Asian populations. These cut‐points were selected to reflect the unique body composition characteristics of Asians, who typically exhibit higher body fat percentage at a given BMI and lower skeletal muscle mass compared with Western populations [14]. Providing thresholds tailored to these population‐specific traits, the JWGSO criteria offer a more appropriate framework for evaluating sarcopenic obesity in Asian individuals, including those with Type 2 diabetes.

In this study, we aimed to evaluate the cross‐sectional association between sarcopenic obesity and diabetic micro‐ and macro‐complications and the longitudinal association between sarcopenic obesity and mortality risk among middle‐aged and older individuals with Type 2 diabetes using the JWGSO criteria suitable for Asian populations.

## 2 Methods

### 2.1 Study Design and Participants

This study utilized data from the KAMOGAWA‐DM cohort. Details of this cohort have been described elsewhere [16]. The study population included individuals with Type 2 diabetes who attended the outpatient endocrinology and diabetology clinics at Kyoto Prefectural University of Medicine Hospital, Kameoka Municipal Hospital and Matsushita Memorial Hospital between January 2015 and May 2024 and underwent both body composition and handgrip strength (HGS) assessments. The diagnosis of Type 2 diabetes was based on the criteria of the American Diabetes Association (ADA). The study protocol was approved by the Ethics Committee of Kyoto Prefectural University of Medicine (approval number: RBMR‐E‐466‐6) and was conducted in accordance with the Declaration of Helsinki and its subsequent revisions. Written informed consent was obtained from all participants. Exclusion criteria were age < 40 years, diagnosis of Type 1 diabetes, impaired glucose tolerance, gestational diabetes, other types of diabetes, absence of Type 2 diabetes diagnosis and lack of HGS or body composition data required for defining sarcopenia and sarcopenic obesity.

### 2.2 Data Collection

The duration of diabetes was determined based on the earliest recorded date among patient self‐report, detection of abnormal laboratory findings or the initiation of diabetes‐specific pharmacological therapy. Lifestyle‐related variables, including smoking status, alcohol consumption and physical activity, were collected from medical records or structured questionnaires. Regular exercise was defined as engaging in any form of physical activity at least once per week. Smoking was defined as current tobacco use, and alcohol consumption as daily intake. Laboratory parameters including haemoglobin A1c (HbA1c), estimated glomerular filtration rate (eGFR) and casual plasma glucose were measured using standard blood tests. HbA1c was reported in National Glycohemoglobin Standardization Program units. Urinary albumin was measured from a spot urine sample and expressed as a ratio to urinary creatinine. Blood pressure values were obtained either from home blood pressure measurements taken in the early morning or from office visits. Hypertension was defined as systolic blood pressure ≥ 140 mmHg, diastolic blood pressure ≥ 90 mmHg or the use of antihypertensive medications. Hyperlipidaemia was defined as fasting low‐density lipoprotein cholesterol ≥ 140 mg/dL, high‐density lipoprotein cholesterol < 40 mg/dL, triglycerides ≥ 150 mg/dL or the use of lipid‐lowering medications. HGS was measured bilaterally using a Smedley‐type dynamometer (TKK, Takei Scientific Instruments Co., Niigata, Japan), and the highest value was used for analysis. Body composition parameters including appendicular skeletal muscle mass, body fat percentage, visceral fat area (VFA) and BMI were assessed using a multi‐frequency bioelectrical impedance analyser (InBody 720, 770 or S10, InBody Japan, Tokyo, Japan). Regarding diabetes‐related complications, diabetic retinopathy was defined as the presence of simple diabetic retinopathy or more severe conditions, as diagnosed in collaboration with ophthalmologists. Diabetic nephropathy was defined as macroalbuminuria, with urinary albumin‐to‐creatinine ratio ≥ 300 mg/gCre. Diabetic neuropathy was diagnosed by the primary care physician based on the presence of distal symmetric polyneuropathy or autonomic neuropathy symptoms, following ADA criteria. Macrovascular complications were defined as a documented history of CVD, including ischaemic heart disease, stroke or peripheral arterial disease. Malignancy was defined as a history of epithelial or non‐epithelial malignant neoplasms diagnosed by a physician or as documented in the medical records. Mortality was confirmed using electronic medical records. Participants who transferred to other hospitals or discontinued follow‐up were right censored at the date of their last recorded visit. Follow‐up duration (in months) was defined from baseline assessment to the earliest occurrence of death, last follow‐up, or transfer to another facility. Causes of death were classified as CVD, malignancy (epithelial or non‐epithelial malignancies), or other causes. Definitions of diabetes, comorbidities, and covariates were based on established clinical guidelines and prior literature (see references in the Supporting Information).

### 2.3 Definition of Sarcopenic Obesity

Sarcopenic obesity was defined according to the criteria proposed by the JWGSO [15].

Sarcopenia status was defined as having both HGS < 28 kg and appendicular skeletal muscle mass/BMI < 0.789 in males and having both HGS < 18 kg and appendicular skeletal muscle mass/BMI < 0.512 in females. Other physical function indicators, such as gait speed, were not assessed due to lack of measurement. Obesity status was defined as having BMI ≥ 25 kg/m^2^ and either VFA ≥ 100 cm^2^ or body fat percentage ≥ 20% in males; and having BMI ≥ 25 kg/m^2^ and either VFA ≥ 100 cm^2^ or body fat percentage ≥ 30% in females. Participants meeting both sarcopenia and obesity criteria were classified as having sarcopenic obesity. Furthermore, the participants were divided into four groups: neither sarcopenia nor obesity, sarcopenia alone, obesity alone and sarcopenic obesity.

## 3 Statistical Analysis

All statistical analyses were performed using R Version 4.4.1 (R Foundation for Statistical Computing, Vienna, Austria) within the RStudio environment. A two‐tailed *p*‐value of < 0.05 was considered statistically significant. Baseline characteristics were presented as means ± standard deviations (SD) for continuous variables or as counts and percentages for categorical variables, as appropriate. No missing covariate data were present in the final analytic sample, allowing for a complete case analysis. Participants were categorized into four groups based on the presence or absence of sarcopenia and obesity: neither sarcopenia or obesity, sarcopenia alone, obesity alone and sarcopenic obesity. Logistic regression analyses were conducted to assess the associations between group classification and the prevalence of diabetic complications, including retinopathy, nephropathy, neuropathy and CVD. Odds ratios (ORs) were calculated using the following models: Model 1: crude, Model 2: adjusted for age and sex, and Model 3: adjusted for Model 2 variables plus HbA1c, duration of diabetes, alcohol use, smoking status, exercise habits, hypertension and hyperlipidaemia. Covariates included in the multivariable models were selected a priori based on established clinical relevance and prior evidence demonstrating their associations with sarcopenia, obesity or mortality risk. Age and sex are fundamental determinants of muscle mass, fat distribution and mortality. BMI and diabetes duration influence both sarcopenia progression and cardiometabolic risk. Lifestyle factors such as smoking status, alcohol consumption and physical activity have been shown to affect muscle quality, adiposity and mortality. Hypertension, dyslipidaemia, CVD, cancer, HbA1c and eGFR were included because they represent major cardiometabolic risk factors and potential confounders that may influence both the development of sarcopenic obesity and mortality outcomes in individuals with Type 2 diabetes, as supported by extensive epidemiologic literature. These covariates were selected to minimize confounding and improve the validity of the estimated associations (see references in the Supporting Information).

To examine the association between sarcopenic obesity and all‐cause mortality, Cox proportional hazards models were applied to calculate hazard ratios (HRs). The proportional hazards assumption was evaluated using Schoenfeld residuals. No significant violations of the assumption were detected. Models were adjusted as follows: Model 1: crude, Model 2: adjusted for age and sex and Model 3: adjusted for Model 2 variables plus HbA1c, duration of diabetes, alcohol use, smoking status, exercise habits, hypertension, hyperlipidaemia, CVD and malignancy.

Survival analysis was conducted using the Kaplan–Meier method to compare mortality across the four groups defined by sarcopenia and obesity status. The log‐rank test was used to assess statistical significance between survival curves.

Subgroup analyses stratified by age were also performed by dividing participants into two groups: 40–75 years and > 75 years.

As a sensitivity analysis, participants were classified into two groups based on the presence or absence of sarcopenic obesity, and Cox proportional hazards models were used to evaluate the association with mortality using the same adjustment models. Subgroup analysis and interaction testing by age category were also conducted in this analysis. Because inclusion of malignancy and CVD could introduce over‐adjustment, we conducted an additional analysis in which these variables were omitted from the covariate set in the primary Cox model for mortality. To address potential small sample bias and separation due to the limited number of deaths in the sarcopenic obesity group, we additionally performed sensitivity analyses using Firth's penalized Cox proportional hazards regression. Penalized models were applied to the overall cohort and to the age‐stratified subgroups (40–75 and > 75 years) using the same covariate structure as in the fully adjusted model (Model 3).

## 4 Results

### 4.1 Study Participants' Characteristics

**FIGURE 1:**
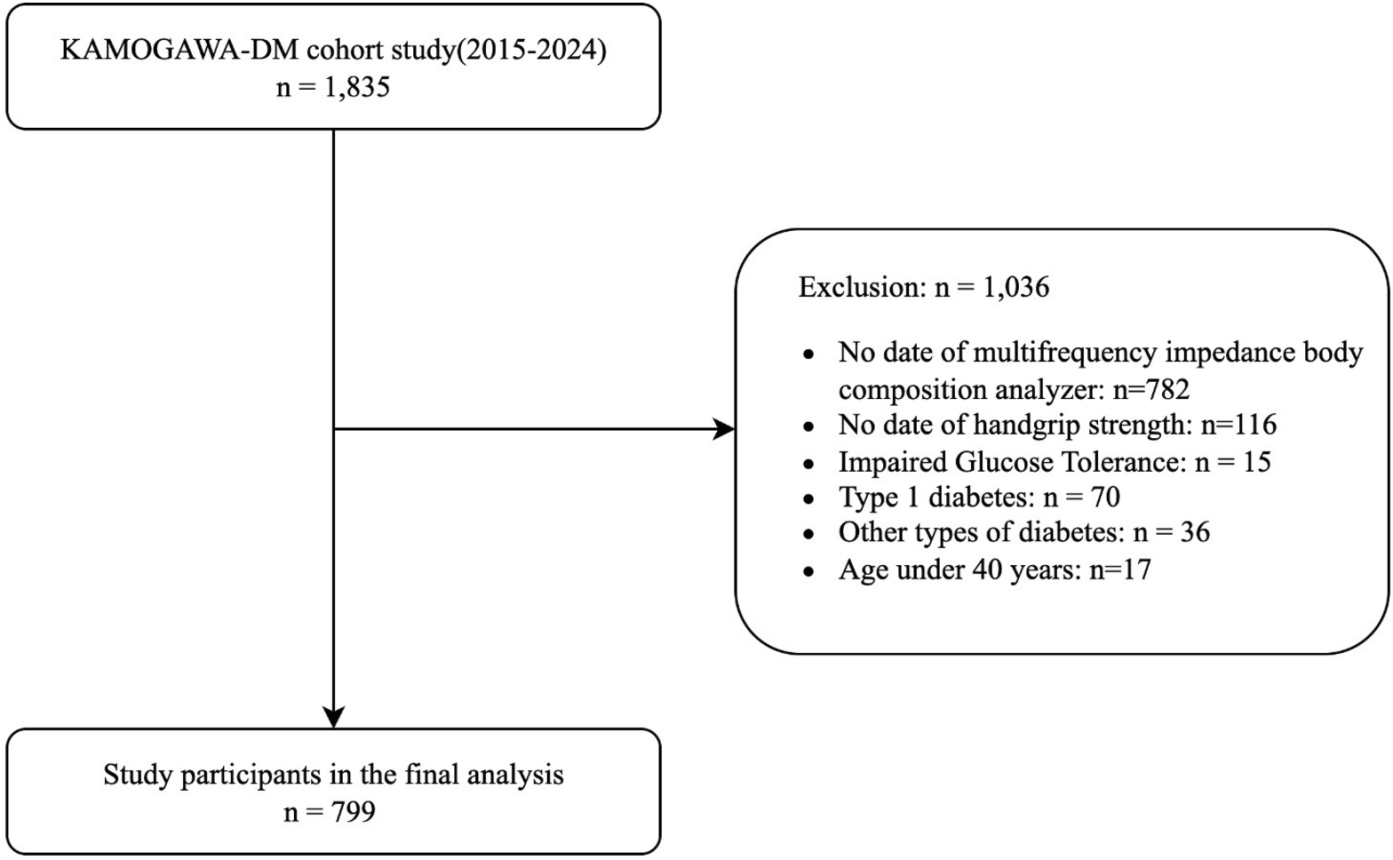
Flow diagram showing the selection of the study population.

From 20 January 2015 to May 2024, a total of 1835 individuals were enrolled in the KAMOGAWA‐DM cohort. After applying the exclusion criteria, 1036 individuals were excluded, and the final analysis included 799 individuals (Figure 1). Among them, 474 were males and 325 were females. The mean age was 68.6 years (SD: 10.4), mean HbA1c was 7.5% (SD: 1.3), and mean BMI was 24.4 kg/m^2^ (SD: 4.4). Participants were categorized into four groups based on the presence or absence of sarcopenia and obesity: neither sarcopenia nor obesity (*n* = 449, 56.2%), sarcopenia alone (*n* = 50, 6.3%), obesity alone (*n* = 276, 34.5%) and sarcopenic obesity (*n* = 24, 3.0%) (Table 1).

**TABLE 1:** Baseline characteristics of the participants.

	All (*N* = 799)	Neither sarcopenia or obesity (*N* = 449)	Obesity alone (*N* = 276)	Sarcopenia alone (*N* = 50)	Sarcopenic obesity (*N* = 24)
Gender (male/female), *n*	474/325	276/173	152/124	37/13	9/15
Age, years	68.6 (10.4)	70.4 (9.3)	63.3 (10.1)	79.0 (6.3)	74.8 (9.7)
HGS, kg	28.2 (9.2)	27.9 (8.7)	30.9 (9.2)	20.6 (6.0)	18.3 (4.1)
ALM, kg	18.3 (4.2)	17.7 (3.9)	20.2 (4.2)	14.5 (3.2)	15.5 (3.0)
BMI, kg/m^2^	24.4 (4.4)	21.8 (2.1)	28.5 (4.0)	21.9 (1.9)	29.8 (3.7)
VFA, cm^2^	96.1 (44.7)	72.9 (25.3)	129.5 (44.6)	90.2 (34.3)	160.1 (47.8)
BFP, %	30.2 (8.8)	25.8 (6.9)	36.0 (7.2)	31.4 (5.8)	44.4 (6.0)
SBP, mmHg	133.3 (18.2)	131.7 (18.9)	135.2 (17.2)	133.9 (16.1)	140.5 (18.4)
DBP, mmHg	75.1 (12.3)	73.2 (11.9)	79.4 (11.7)	68.1 (12.2)	75.3 (12.6)
Glucose, mg/dL	154.6 (51.7)	151.6 (47.1)	153.1 (50.1)	188.7 (79.8)	156.3 (57.7)
Haemoglobin A1c, %	7.5 (1.3)	7.5 (1.3)	7.6 (1.3)	7.6 (1.1)	7.5 (1.8)
LDL‐C, mg/dL	108.0(29.7)	107.5 (31.2)	110.2 (28.1)	102.9 (24.6)	103.9 (28.1)
HDL‐C, mg/dL	58.1 (16.8)	60.8 (17.5)	54.8 (15.8)	54.1 (14.2)	54.6 (10.2)
TG, mg/dL	140.7 (89.2)	125.5 (83.1)	167.5 (99.0)	121.8 (45.6)	154.3 (76.2)
eGFR, mL/min/1.73 m^2^	69.2 (22.0)	69.1 (21.5)	71.8 (21.1)	60.3 (23.5)	59.5 (30.2)
Duration, years	15.5 (11.1)	17.1 (11.3)	11.9 (9.1)	21.5 (13.7)	15.5 (9.8)
Retinopathy (*N* = 657), *n*	171 (26.0)	102.0 (27.8)	53.0 (23.3)	13.0 (31.7)	3.0 (13.6)
Nephropathy (*N* = 674), *n*	91 (13.5)	47.0 (12.6)	25.0 (10.5)	10.0 (23.8)	9.0 (40.9)
Neuropathy (*N* = 651), *n*	200 (30.7)	110.0 (30.1)	62.0 (27.3)	21.0 (55.3)	7.0 (33.3)
CVD, *n*	140 (17.5)	74.0 (16.5)	50.0 (18.1)	8.0 (16.0)	8.0 (33.3)
Death, *n*	41 (5.1)	24.0 (5.3)	4.0 (1.4)	8.0 (16.0)	5.0 (20.8)
Exercise, *n*	315 (39.4)	194.0 (43.2)	97.0 (35.1)	16.0 (32.0)	8.0 (33.3)
Alcohol habit, *n*	395 (49.4)	237.0 (52.8)	127.0 (46.0)	18.0 (36.0)	13.0 (54.2)
Smoking status, *n*	249 (31.2)	138.0 (30.7)	85.0 (30.8)	22.0 (44.0)	4.0 (16.7)
Hypertension, *n*	461 (57.7)	240.0 (53.5)	168.0 (60.9)	33.0 (66.0)	20.0 (83.3)
Hyperlipidaemia, *n*	456 (57.1)	251.0 (55.9)	165.0 (59.8)	28.0 (56.0)	12.0 (50.0)
Cancer, *n*	121(15.1)	64(14.3)	42(15.2)	9(18.0)	6(25.0)

*Note:* Data were expressed as mean (standard deviation) or number (%). Participants were classified into four groups: No sarcopenic no obesity, no sarcopenic obesity, sarcopenic no obesity and sarcopenic obesity.

Abbreviations: ALM = appendicular lean mass, BFP = body fat percentage, BMI = body mass index, CVD = cardiovascular disease, DBP = diastolic blood pressure, eGFR = estimated glomerular filtration rate, HDL‐C = high‐density lipoprotein cholesterol, HGS = handgrip strength, LDL‐C = low‐density lipoprotein cholesterol, SBP = systolic blood pressure, TG = triglycerides, VFA = visceral fat area.

### 4.2 Association Between Sarcopenic Obesity and Diabetic Micro‐ and Macro‐Complications

Multivariable logistic regression analysis was performed to assess the association between sarcopenic obesity and diabetic micro‐ and macro‐complications. After adjusting for potential confounders, the sarcopenic obesity group showed a significantly increased odds ratio for prevalence of nephropathy compared with the neither sarcopenia nor obesity group (adjusted OR: 3.55, 95% CI: 1.36–9.30, *p* = 0.01) (Table 2). Furthermore, although it did not reach statistical significance, the sarcopenic obesity group tended to be increased odds ratio for the prevalence of CVD compared with the neither sarcopenia nor obesity group (adjusted OR: 2.46, 95% CI: 0.99–6.09, *p* = 0.051).

**TABLE 2:** Association between sarcopenic obesity and diabetic complications.

	Group	Model 1		Model 2		Model 3	
		OR (95% CI)	*p*	OR (95% CI)	*p*	OR (95% CI)	*p*
Retinopathy	Neither sarcopenia or obesity	Ref		Ref		Ref	
	Obesity alone	0.79 (0.54–1.16)	0.23	0.94 (0.63–1.41)	0.77	0.94 (0.61–1.46)	0.79
	Sarcopenia alone	1.21 (0.60–2.42)	0.60	0.98 (0.48–2.02)	0.96	0.75 (0.35–1.63)	0.47
	Sarcopenic obesity	0.41 (0.12–1.42)	0.16	0.34 (0.10–1.18)	0.09	0.27 (0.07–1.01)	0.052
Nephropathy	Neither sarcopenia or obesity	Ref		Ref		Ref	
	Obesity alone	0.81 (0.48–1.36)	0.43	1.07 (0.62–1.85)	0.80	0.91 (0.52–1.62)	0.76
	Sarcopenia alone	2.16 (1.00–4.68)	0.051	1.50 (0.67–3.35)	0.33	1.42 (0.62–3.24)	0.40
	Sarcopenic obesity	4.79 (1.94–11.8)	<0.001	4.37 (1.72–11.1)	0.002	3.55 (1.36–9.30)	0.01
Neuropathy	Neither sarcopenia or obesity	Ref		Ref		Ref	
	Obesity alone	0.87 (0.60–1.26)	0.46	1.09 (0.74–1.62)	0.66	1.10 (0.73–1.67)	0.63
	Sarcopenia alone	2.86 (1.45–5.64)	0.002	2.21 (1.10–4.45)	0.03	1.95 (0.94–4.02)	0.07
	Sarcopenic obesity	1.16 (0.46–2.95)	0.76	0.92 (0.36–2.40)	0.87	0.86 (0.32–2.28)	0.76
CVD	Neither sarcopenia or obesity	Ref		Ref		Ref	
	Obesity alone	1.12 (0.76–1.66)	0.57	1.17 (0.77–1.77)	0.47	1.14 (0.74–1.75)	0.55
	Sarcopenia alone	0.97 (0.44–2.14)	0.93	0.92 (0.41–2.07)	0.83	0.93 (0.41–2.12)	0.87
	Sarcopenic obesity	2.53 (1.05–6.14)	0.04	2.52 (1.03–6.17)	0.04	2.46 (0.99–6.09)	0.051

*Note:* Logistic regression analyses were performed to examine the associations of sarcopenic obesity and its components with the presence of diabetic complications, including retinopathy, nephropathy, neuropathy and cardiovascular disease. Model 1 is a crude model without adjustment. Model 2 is adjusted for age and sex. Model 3 is further adjusted for diabetes duration, haemoglobin A1c, exercise habits, alcohol consumption, smoking status, hypertension and hyperlipidaemia.

Abbreviations: CI = confidence interval, CVD = cardiovascular disease, OR = odds ratio, Ref = reference group.

### 4.3 Impact of Sarcopenic Obesity on Mortality

During the follow‐up period (median: 46 months), 41 participants died (Table S3).

**FIGURE 2:**
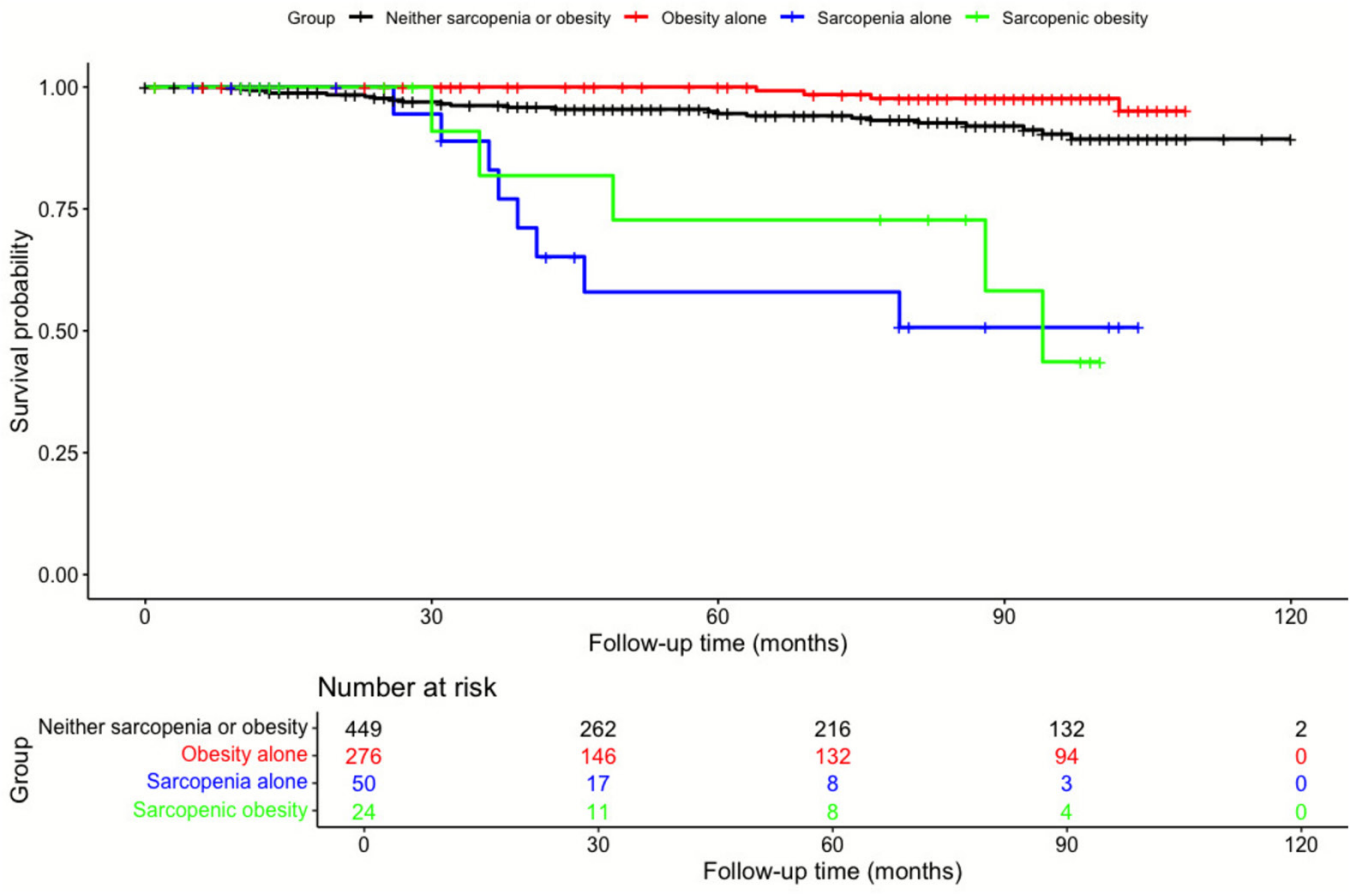
Kaplan–Meier survival curves for all‐cause mortality stratified by sarcopenic and obese status. Cox proportional hazards regression (Model 3, fully adjusted) showed: reference = neither sarcopenia or obesity; obesity alone HR 0.53 (95% CI 0.18–1.57, *p* = 0.25); sarcopenia alone HR 2.36 (0.99–5.60, *p* = 0.053); sarcopenic obesity HR 2.89 (1.01–8.30, *p* = 0.048). Total number of events: 41 (neither sarcopenia or obesity = 24, obesity alone = 4, sarcopenia alone = 8, sarcopenic obesity = 5).

Cox proportional hazards models were used to evaluate the association between each group and all‐cause mortality. Kaplan–Meier survival analysis with log‐rank testing revealed a significant difference in survival among the four groups (*p* < 0.001) (Figure 2). In the multivariable‐adjusted model, sarcopenic obesity groups (adjusted HR: 2.89, 95% CI: 1.01–8.30, *p* = 0.048) had significantly higher HRs compared to the neither sarcopenia nor obesity group (Table 3).

**TABLE 3:** Association between sarcopenic obesity and all‐cause mortality.

Group	Model 1		Model 2		Model 3	
	HR (95% CI)	*p*	HR (95% CI)	*p*	HR (95% CI)	*p*
Neither sarcopenia or obesity	Ref		Ref		Ref	
Obesity alone	0.27 (0.09–0.77)	0.015	0.50 (0.17–1.46)	0.20	0.53 (0.18–1.57)	0.25
Sarcopenia alone	6.40 (2.85–14.4)	< 0.001	2.80 (1.20–6.50)	0.017	2. 36 (0.99–5.6)	0.053
Sarcopenic obesity	5.34 (2.03–14.0)	< 0.001	2.75 (1.02–7.44)	0.047	2.89 (1.01–8.30)	0.048

*Note:* Cox proportional hazards regression analyses were performed to examine the associations of sarcopenic obesity and its components with all‐cause mortality. Model 1 is a crude model without adjustment. Model 2 is adjusted for age and sex. Model 3 is further adjusted for diabetes duration, haemoglobin A1c, exercise habits, alcohol consumption, smoking status, hypertension, hyperlipidaemia, cardiovascular disease and cancer.

Abbreviations: CI = confidence interval, HR = hazard ratio, Ref = reference group.

Subgroup analysis by age group (40–75 years and > 75 years) revealed age‐specific patterns. In participants aged 40–75 years, the sarcopenic obesity group exhibited a markedly increased HR (adjusted HR: 13.1, 95% CI: 2.93–58.4, *p* < 0.001), whereas in those aged > 75 years, the sarcopenia alone group (adjusted HR: 3.21, 95% CI: 1.07–8.33, *p* = 0.04), but the sarcopenic obesity group (adjusted HR [0.34–8.81]) showed a significant increase in mortality risk (Table 4 and Figure S1 and S2).

**TABLE 4:** Subgroup analysis of the association between sarcopenic obesity and all‐cause mortality.

	Group	Model 1		Model 2		Model 3	
		HR (95% CI)
*p*	HR (95% CI)	*p*	HR (95% CI)	*p*
Age 4075 years (*N* = 590)	Neither sarcopenia or obesity	Ref		Ref		Ref	
	Obesity alone	0.50 (0.16–1.59)	0.24	0.51 (0.16–1.61)	0.25	0.57 (0.17–1.83)	0.34
	Sarcopenia alone	3.31 (0.43–25.7)	0.25	3.33 (0.43–26.0)	0.25	4.41 (0.47–41.7)	0.20
	Sarcopenic obesity	11.3 (3.12–40.7)	< 0.001	12.6 (3.44–45.9)	< 0.001	13.1 (2.93–58.4)	< 0.001
Age > 75 years (*N* = 209)	Neither sarcopenia or obesity	Ref		Ref		Ref	
	Obesity alone	0.00 (0.00–Inf)	0.99	0.00 (0.00–Inf)	0.99	0.00 (0.00–Inf)	0.99
	Sarcopenia alone	3.21 (1.27–8.13)	0.01	3.07 (1.21–7.80)	0.02	3.21 (1.07–8.33)	0.04
	Sarcopenic obesity	1.35 (0.30–6.00)	0.70	1.45 (0.32–6.5)	0.63	1.41 (0.34–8.81)	0.51

*Note:* Cox proportional hazards regression analyses were performed to examine the associations of sarcopenic obesity and its components with all‐cause mortality in subgroups stratified by age (40–75 years and over 75 years). Model 1 is a crude model without adjustment. Model 2 is adjusted for sex. Model 3 is further adjusted for diabetes duration, haemoglobin A1c, exercise habits, alcohol consumption, smoking status, hypertension, hyperlipidaemia, cardiovascular disease and cancer.

Abbreviations: CI = confidence interval, HR = hazard ratio, Ref = reference group.

**FIGURE 3:**
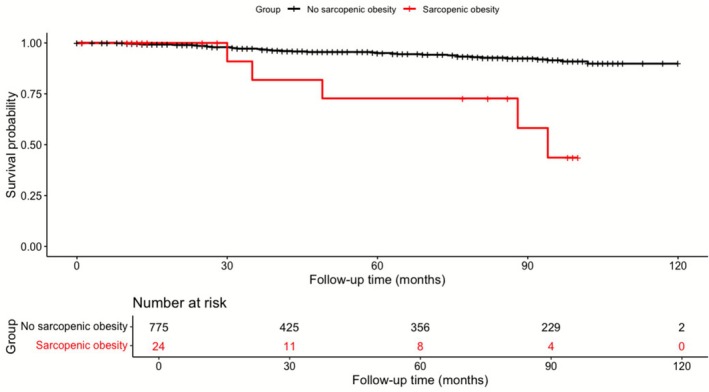
Kaplan–Meier survival curves for all‐cause mortality stratified by sarcopenic obesity status. Cox proportional hazards regression (Model 3, fully adjusted) showed: sarcopenic obesity (+) HR 4.0 (95% CI 1.44–11.0, *p* = 0.008) versus sarcopenic obesity (−). Total number of events: 41 (sarcopenic obesity (−) = 36, sarcopenic obesity (+) = 5).

For sensitivity analysis, participants were dichotomized into sarcopenic obesity and non‐sarcopenic obesity groups, and all‐cause mortality was assessed using a Cox proportional hazards model. Kaplan–Meier analysis demonstrated a significant survival difference between the two groups (log‐rank *p* < 0.001) (Figure 3). The sarcopenic obesity group had a significantly higher HR for mortality (adjusted HR: 4.0, 95% CI: 1.44–11.0, *p* = 0.008). In age‐stratified analysis, this association remained significant in participants aged 40–75 years (adjusted HR: 14.1, 95% CI: 3.25–61.5, *p* < 0.001), but not in those aged > 75 years (adjusted HR: 1.55, 95% CI: 0.32–7.46, *p =* 0.59) (interaction *p* = 0.03) (Table 5).

**TABLE 5:** Association between sarcopenic obesity and all‐cause mortality.

	Group	Model 1		Model 2		Model 3		*p* for interaction
		HR (95% CI)	*p*	HR (95% CI)	*p*	HR (95% CI)	*p*	
All								
	Sarcopenic obesity (−)	Ref		Ref		Ref		
	Sarcopenic obesity (+)	5.87 (2.30–15.0)	< 0.001	3.66 (1.40–9.55)	0.008	4.0 (1.44–11.0)	0.008	
Age 40–75 years (*N* = 590)								0.03
	Sarcopenic obesity (−)	Ref		Ref		Ref		
	Sarcopenic obesity (+)	13.5 (3.91–46.8)	< 0.001	15.0 (4.29–52.5)	< 0.001	14.1 (3.25–61.5)	<0.001	
Age > 75 years (*N* = 209)								
	Sarcopenic obesity (−)	Ref		Ref		Ref		
	Sarcopenic obesity (+)	1.23 (0.29–5.30)	0.78	1.36 (0.31–5.90)	0.68	1.55 (0.32–7.46)	0.59	

*Note:* Cox proportional hazards regression analyses were performed to examine the association between the presence of sarcopenic obesity and all‐cause mortality in the overall population and by age group (40–75 years and > 75 years). Model 1 is a crude model without adjustment. Model 2 is adjusted for age and sex. Model 3 is further adjusted for diabetes duration, haemoglobin A1c, exercise habits, alcohol consumption, smoking status, hypertension, hyperlipidaemia, cardiovascular disease and cancer.

Abbreviations: CI = confidence interval, HR = hazard ratio, Ref = reference group.

When malignancy and CVD were excluded from the adjustment set, the results remained materially unchanged. The aHRs were 0.53 (95% CI 0.18–1.56, *p* = 0.25) for the obesity‐only group, 2.46 (95% CI 1.04–5.77, *p* = 0.04) for the sarcopenia‐only group and 2.91 (95% CI 1.02–8.31, *p* = 0.046) for the sarcopenic obesity group, indicating that removal of these comorbidities from the model did not alter the overall pattern of associations (Table S4).

Using Firth's penalized Cox regression, the direction and approximate magnitude of the associations between sarcopenic obesity categories and all‐cause mortality remained generally consistent with those obtained from the conventional Cox models. Although confidence intervals were wide due to the limited number of events, the overall conclusions were unchanged. Detailed estimates from the penalized models are presented in Table S4.

## 5 Discussion

In this study, we cross‐sectionally evaluated the association between sarcopenic obesity and diabetic micro‐ and macro‐complications, revealing that there is a relationship between sarcopenic obesity and the prevalence of nephropathy and CVD and longitudinally assessed the impact of sarcopenic obesity on mortality risk, revealing that sarcopenic obesity is associated with mortality risk, especially in individuals aged 40–75 years. This is the first study to employ the diagnostic criteria for sarcopenic obesity recently proposed for Asian populations and to investigate the relationship between sarcopenic obesity and diabetic micro‐ and macro‐complications and mortality risk.

Due to differing definitions in the literature, the reported prevalence of sarcopenic obesity varies widely. However, studies that defined obesity using BMI reported a prevalence of 2.8% [13], which is consistent with the prevalence observed in our study. Our findings showed that sarcopenic obesity was significantly associated with the prevalence of nephropathy. Previously, we reported a significant association between sarcopenic obesity and macroalbuminuria [17], although the diagnostic criteria used at that time differed from those used in the current study. Nevertheless, a similar trend was observed. Although there was no statistically significant association, sarcopenic obesity tended to be associated with the presence of macrovascular complications, which is partly consistent with previous reports [7].

A meta‐analysis evaluating the association between sarcopenia and renal function or urinary albumin in individuals with diabetes reported that sarcopenia was significantly associated with increased urinary albumin levels and decreased eGFR [18]. Several studies, including those conducted in Japan [19], have reported on the association between sarcopenic obesity and decreased renal function. Importantly, sarcopenic obesity has been shown to pose a greater risk of renal function decline than sarcopenia without obesity. This may be explained by the chronic inflammation and increased insulin resistance associated with sarcopenia [20] as well as adverse renal haemodynamic effects such as glomerular hypertrophy and hyperfiltration due to fat accumulation, which may directly impair kidney function [21]. Consistent with previous findings, our study demonstrated a significant association between diabetic nephropathy and sarcopenic obesity, but not with sarcopenia or obesity alone.

Regarding mortality risk, the presence of sarcopenic obesity was significantly associated with increased mortality. And age‐stratified analysis revealed differing trends: among individuals aged ≤ 75 years, those with both sarcopenic obesity had a significantly increased mortality risk. Conversely, among individuals aged > 75 years, sarcopenia without obesity was significantly associated with higher mortality risk. Sensitivity analyses showed similar trends. However, these trends should be interpreted with careful consideration of statistical uncertainty. In this study, the number of individuals with sarcopenic obesity (*n* = 24) and the number of deaths (*n* = 41) were limited, resulting in wide confidence intervals for the estimated HRs, particularly in the age‐stratified analyses where imprecision was more pronounced. For example, in the 40–75‐year group, the adjusted HR for sarcopenic obesity was 13.1, whereas the 95% CI ranged from 2.93 to 58.4, making it difficult to draw definitive conclusions regarding the magnitude of the true effect. Such a wide confidence interval indicates that, although the direction of the association (increased risk) appears relatively consistent, the effect size is estimated with low precision, necessitating cautious interpretation in clinical contexts. The instability inherent to rare events means that small variations between groups can lead to substantial fluctuations in the HR estimate, and this uncertainty directly affects the overall reliability of the results.

A previous report showed sarcopenic obesity was associated with increased mortality risk, but sarcopenia without obesity was not associated with increased mortality risk. In contrast, our findings showed that sarcopenia without obesity was also associated with increased mortality risk, especially individuals aged > 75 years [13]. In contrast, our findings indicate that sarcopenia without obesity was also linked to increased mortality among individuals aged > 75 years. This discrepancy may be partly explained by differences in study populations, particularly the higher mean age in our cohort. This discrepancy is of particular interest and may reflect age‐related differences. Although prior studies targeted individuals aged ≥ 60 years (with a median age of 66–67.5 years), our study included participants aged ≥ 40 years, with a higher mean age of 68.6 years. This suggests that our cohort may include a relatively older population, making simple comparisons difficult. Notably, the subgroup aged 40–75 years showed results consistent with a previous study. Notably, the subgroup aged 40–75 years showed results consistent with previous studies. Because the diagnostic criteria for sarcopenic obesity proposed by the JWSGO target individuals aged 40–75 years, the influence of sarcopenic obesity should be interpreted with consideration of age.

In the group aged > 75 years, no deaths occurred in the obesity‐only category, leading to unstable HR estimation in the Cox regression model and resulting in an HR of 0 due to statistical constraints. This situation reflects complete separation, in which the confidence interval theoretically extends from zero towards infinity because no events were observed in one subgroup. In parallel, survival bias may also contribute to this pattern; individuals with obesity who survived to advanced age may represent a relatively healthier subset, which could attenuate the observed association between obesity and mortality. Previous studies have shown that, among older adults, the effect of obesity on mortality risk tends to diminish, whereas the influence of malnutrition and sarcopenia becomes more prominent, often described as the obesity paradox [22, 23]. Taken together, the observation that sarcopenia was more strongly associated with mortality than sarcopenic obesity among individuals aged > 75 years likely reflects both statistical instability arising from zero events and underlying epidemiologic characteristics specific to the very old population. Overall, these observations suggest that among individuals aged > 75 years, the impact of sarcopenia on decreased muscle mass and strength may be more prominent than the influence of obesity. These findings underscore the need for further research, including reconsideration of obesity definitions (e.g., based on BMI), in this age group.

Beyond age related differences, potential racial differences should also be considered when interpreting our findings. Several studies conducted in Western populations have, as noted earlier, reported weaker or inconsistent associations between sarcopenic obesity and mortality [24]. These discrepancies may be partly explained by well‐established ethnic variations in body composition. Asians typically exhibit lower skeletal muscle mass, higher body fat percentage and greater visceral adiposity at a given BMI than individuals of European descent, along with a stronger propensity towards insulin resistance [25]. Such physiological characteristics may amplify the adverse consequences of sarcopenic obesity in Asian populations [26, 27]. Taken together, these considerations indicate that ethnic‐specific criteria and population‐specific physiological characteristics are essential when evaluating the impact of sarcopenic obesity on mortality.

In addition, this study did not measure key indicators of nutritional status such as serum albumin levels or dietary intake nor biomarkers of inflammation, including C‐reactive protein. The absence of these variables raises the possibility of unmeasured confounding. Chronic inflammation and malnutrition frequently accompany sarcopenia and sarcopenic obesity, and these conditions are known to be strongly associated with mortality as well as renal and cardiovascular outcomes [28, 29, 30, 31]. Failure to adjust for these factors may have biased our findings in either direction: The risk associated with sarcopenic obesity may have been overestimated if adverse health conditions clustered in this group, whereas the effect of obesity may have been underestimated if inflammation or malnutrition was more prevalent among individuals classified as obese. Furthermore, we were unable to assess hormonal markers such as testosterone or IGF‐1, which play an important role in muscle metabolism and may contribute to the biological mechanisms linking sarcopenic obesity with adverse outcomes [32, 33]. Because inflammation, malnutrition and loss of muscle mass are closely interrelated in older adults and in individuals with diabetes [34], these unmeasured factors could also partially mediate the observed association between sarcopenic obesity and mortality. The absence of these mechanistic biomarkers limits the extent to which biological pathways can be inferred, underscoring the need for future longitudinal studies incorporating comprehensive assessments of nutritional, inflammatory and hormonal status.

Building on these mechanistic insights, several biological mechanisms may help explain the elevated mortality risk observed in individuals with sarcopenic obesity. Both excess adiposity and loss of skeletal muscle contribute to a chronic low‐grade inflammatory state characterized by increased CRP, IL‐6 and TNF‐α [35, 36]. Obesity promotes secretion of pro‐inflammatory adipokines (e.g., leptin and resistin) while suppressing anti‐inflammatory adiponectin, whereas sarcopenia reduces myokines such as irisin and IL‐15 that normally exert protective metabolic and anti‐inflammatory effects [37, 38]. This imbalance between adipokines and myokines may exacerbate systemic inflammation, insulin resistance, endothelial dysfunction and catabolic pathways. Furthermore, metabolic crosstalk between adipose and muscle tissue including ectopic fat accumulation, mitochondrial dysfunction and impaired glucose and lipid metabolism may contribute to increased vulnerability to adverse outcomes [39]. These mechanistic pathways provide a plausible biological basis supporting the epidemiologic associations observed in this study.

## 6 Limitations

Several limitations of this study should be noted. First, the cohort consisted exclusively of Japanese individuals, potentially limiting the generalizability of the findings to other populations. Although the JWGSO diagnostic criteria used in this study were specifically developed for Asian populations, substantial ethnic variation still exists within Asia in terms of body composition, fat distribution, muscle mass and metabolic characteristics [25]. These differences may influence both the prevalence and prognostic impact of sarcopenic obesity. Caution is warranted when extrapolating our findings even to other Asian populations. Validation of the JWGSO criteria and of the age‐dependent mortality patterns observed in this study in multiethnic and international cohorts will be essential to clarify whether these associations reflect universal mechanisms or population‐specific characteristics. Second, the association between sarcopenic obesity and diabetic micro‐ and macrovascular complications was evaluated in a cross‐sectional manner. Because exposure and outcome were measured at the same time point, temporal ordering could not be established, and reverse causation cannot be ruled out. Additionally, unmeasured confounding related to disease duration, glycaemic fluctuations or prior metabolic decline may have influenced these cross‐sectional associations. These analyses should not be interpreted as evidence of causality. Longitudinal studies with repeated measurements of both sarcopenic obesity status and vascular outcomes will be essential to clarify the temporal and causal pathways linking these conditions. Third, given the limited number of deaths and the small number of participants diagnosed with sarcopenic obesity, the statistical power may have been insufficient, as indicated by the wide confidence intervals. As a result, the effect of sarcopenic obesity on cardiovascular‐ or malignancy‐related mortality could not be determined with certainty. Because individuals with pre‐existing malignancies were not excluded at cohort enrollment, the potential influence of these conditions on mortality outcomes should be acknowledged. Fourth, we were unable to assess gait speed or the chair stand test, both of which are recommended as key functional components in the EWGSOP2 criteria [40]. The absence of these performance‐based measures may limit comparability with studies adopting the EWGSOP2 framework and may have influenced the accuracy of functional assessment in diagnosing sarcopenia. Although the JWGSO criteria used in this study do not require gait speed or chair stand tests and were specifically developed for Asian populations, the lack of these measures should still be acknowledged as a limitation when interpreting our findings in relation to international diagnostic standards. Fifth, potential confounders such as nutritional status and inflammatory markers were not measured and could not be adjusted for. Lastly, the diagnostic criteria for sarcopenic obesity used in this study may not be appropriate for individuals over 75 years of age, warranting the development of new age‐specific criteria.

## 7 Conclusion

This study examined the association between sarcopenic obesity and both diabetic micro‐ and macro‐complications and all‐cause mortality in middle‐aged and older individuals with Type 2 diabetes, using diagnostic criteria developed by the JWGSO for Asian populations. Sarcopenic obesity was significantly linked to diabetic nephropathy, whereas sarcopenia or obesity alone were not. Additionally, sarcopenia—regardless of obesity—was associated with higher mortality. Notably, sarcopenic obesity markedly increased mortality risk in individuals aged 40–75 years, whereas in those aged > 75 years, sarcopenia in the absence of obesity was associated with an increased risk, rather than sarcopenic obesity. These findings highlight the age‐specific impact of sarcopenic obesity and support the clinical utility of the JWGSO criteria. Future studies should refine diagnostic thresholds for older populations and clarify causal mechanisms through longitudinal research.

## Funding

The authors have nothing to report.

## Ethics Statement

The ethics committee approved the study (approval number: ERB‐C‐1876; approval date: 27/11/2020), which was conducted in accordance with the Declaration of Helsinki. Written informed consent was obtained from all participants prior to their inclusion in the study.

## Consent

The authors have nothing to report.

## Conflicts of Interest

Yoshitaka Hashimoto received personal fees from Novo Nordisk Pharma Ltd., Sanofi K.K., Sumitomo Dainippon Pharma Co. Ltd., Nippon Boehringer Ingelheim Co., Mitsubishi Tanabe Pharma Corp., Kowa Company Ltd., Taisho Pharma Co., Eli Lilly Japan K.K. and Daiichi Sankyo Co. Hiroshi Okada received grants from the Japan Diabetes Foundation and received personal fees from Mochida Pharma Co. Ltd., Teijin Pharma Ltd., MSD K.K., Mitsubishi Tanabe Pharma Corporation, AstraZeneca K.K., Sumitomo Dainippon Pharma Co. Ltd., Novo Nordisk Pharma Ltd., Daiichi Sankyo Co. Ltd., Eli Lilly Japan K.K., Kyowa Hakko Kirin Company Ltd., Kissei Pharmaceutical Co. Ltd., Takeda Pharmaceutical Co. Ltd., Kowa Pharmaceutical Co. Ltd., Ono Pharmaceutical Co. Ltd. and Sanofi K.K. Emi Ushigome received grant support from the Japanese Study Group for Physiology and Management of Blood Pressure, the Astellas Foundation for Research on Metabolic Disorders (Grant Number 4024) and Mishima Kaiun Memorial Foundation and received personal fees from Nippon Boehringer Ingelheim Co. Ltd., Mitsubishi Tanabe Pharma Corporation, Daiichi Sankyo Co. Ltd., MSD K.K., Kyowa Hakko Kirin Co. Ltd., Sumitomo Dainippon Pharma Co. Ltd., Kowa Pharmaceutical Co. Ltd., Novo Nordisk Pharma Ltd., Ono Pharmaceutical Co. Ltd., Taisho Pharmaceutical Co. Ltd. and Sanofi K.K., outside the submitted work. Donated Fund Laboratory of Diabetes therapeutics is an endowment department, supported with an unrestricted grant from Ono Pharmaceutical Co. Ltd., Taiyo Kagaku Co. Ltd. and Taisho Pharmaceutical Co. Ltd. Masahide Hamaguchi received grants from AstraZeneca K.K., Ono Pharma Co. Ltd. and Kowa Pharma Co. Ltd. and received personal fees from AstraZeneca K.K., Ono Pharma Co. Ltd., Eli Lilly, Japan, Sumitomo Dainippon Pharma Co. Ltd., Daiichi Sankyo Co. Ltd., Mitsubishi Tanabe Pharma Corp., Sanofi K.K. and Kowa Pharma Co. Ltd. outside of the submitted work. Hanako Nakajima received personal fees from Kowa Pharmaceutical Co. Ltd., Kyowa Hakko Kirin Co. Ltd. and Nippon Boehringer Ingelheim Co. Ltd. Takafumi Osaka received personal fees from Nippon Boehringer Ingelheim Co. Ltd., Mitsubishi Tanabe Pharma Corp., Daiichi Sankyo Co. Ltd., Sanofi K.K., Takeda Pharma Co. Ltd., MSD K.K., Sumitomo Dainippon Pharma Co. Ltd., Kowa Pharma Co. Ltd., Novo Nordisk Pharma Ltd., Ono Pharma Co. Ltd., Eli Lilly Japan K.K., Taisho Pharma Co. Ltd., AstraZeneca K.K., Abbott Japan Co. Ltd., Teijin Pharma Ltd., Medtronic Japan Co. Ltd., Otsuka Pharma Co. Ltd. and TERUMO CORPORATION, outside the submitted work. Naoko Nakanishi received personal fees from Kowa Pharmaceutical Co. Ltd. and Novo Nordisk Pharma Ltd., Nippon Boehringer Ingelheim Co. Ltd. and TERUMO CORPORATION. Michiaki Fukui received grants from Ono Pharma Co. Ltd., Oishi Kenko inc., Yamada Bee Farm, Nippon Boehringer Ingelheim Co. Ltd., Kissei Pharma Co. Ltd., Mitsubishi Tanabe Pharma Corp., Daiichi Sankyo Co. Ltd., Sanofi K.K., Takeda Pharma Co. Ltd., Astellas Pharma Inc., MSD K.K., Kyowa Kirin Co. Ltd., Sumitomo Dainippon Pharma Co. Ltd., Kowa Pharma Co. Ltd., Novo Nordisk Pharma Ltd., Sanwa Kagagu Kenkyusho CO. Ltd., Eli Lilly, Japan, K.K., Taisho Pharma Co. Ltd., Terumo Corp., Tejin Pharma Ltd., Nippon Chemiphar Co. Ltd., Abbott Japan Co. Ltd., Johnson & Johnson K.K. Medical Co. and TERUMO CORPORATION and received personal fees from Nippon Boehringer Ingelheim Co. Ltd., Kissei Pharma Co. Ltd., Mitsubishi Tanabe Pharma Corp., Daiichi Sankyo Co. Ltd., Sanofi K.K., Takeda Pharma Co. Ltd., Astellas Pharma Inc., MSD K.K., Kyowa Kirin Co. Ltd., Sumitomo Dainippon Pharma Co. Ltd., Kowa Pharma Co. Ltd., Novo Nordisk Pharma Ltd., Ono Pharma Co. Ltd., Sanwa Kagaku Kenkyusho Co. Ltd., Eli Lilly Japan K.K., Taisho Pharma Co. Ltd., Bayer Yakuhin Ltd., AstraZeneca K.K., Mochida Pharma Co. Ltd., Abbott Japan Co. Ltd., Teijin Pharma Ltd., Arkray Inc., Medtronic Japan Co. Ltd. and Nipro Corp., TERUMO CORPORATION, outside the submitted work. The other authors declare no conflicts of interest.

## Supporting information


**Data S1:** Supporting Information.

**Table S1:** Baseline characteristics of the participants in individuals aged 40–75 years.
**Table S2:** Baseline characteristics of the participants in individuals aged over 75 years.
**Table S3:** The association between sarcopenic obesity and all‐cause mortality after multivariable adjustment.
**Table S4:** Firth's penalized Cox regression for the association between sarcopenic obesity and all‐cause Mortality
**Table S5:** Distribution of causes of death according to sarcopenic obesity group.

**Figure S1:** Kaplan–Meier survival curves for all‐cause mortality stratified by sarcopenic and obese status in individuals aged 40–75 years. Cox proportional hazards regression (Model 3, fully adjusted) showed: reference = neither sarcopenia or obesity; obesity alone HR 0.57 (95% CI 0.17–1.83, *p* = 0.34); sarcopenia alone HR 4.41 (0.47–41.7, *p* = 0.20); sarcopenic obesity HR 13.1 (2.93–58.4, *p* < 0.001). Total number of events: 19 (neither sarcopenia or obesity = 11, obesity alone = 4, sarcopenia alone = 1, sarcopenic obesity = 3).

**Figure S2:** Kaplan–Meier survival curves for all‐cause mortality stratified by sarcopenic and obese status in individuals aged over 75 years. Cox proportional hazards regression (Model 3, fully adjusted) showed: reference = neither sarcopenia or obesity; obesity alone HR 0.00 (95% CI 0.00–Inf, *p* = 0.99); sarcopenia alone HR 3.21 (1.07–8.33, *p* = 0.04); sarcopenic obesity HR 1.41 (0.34–8.81, *p* = 0.51). Total number of events: 22 (neither sarcopenia or obesity = 13, obesity alone = 0, sarcopenia alone = 7, sarcopenic obesity = 2).

## Data Availability

Data from this study are available from the corresponding author upon reasonable request.
