# Supplementary material for: Age‐Stratified Associations of Sarcopenic Obesity With Mortality in Type 2 Diabetes
Source: J Cachexia Sarcopenia Muscle. 2026 Jan 28;17(1):e70211. doi: 10.1002/jcsm.70211 (PMC12848597; doi:10.1002/jcsm.70211)
Supplement: Supplementary file 1 — Data S1: Supporting Information. [file JCSM-17-e70211-s003.docx]

**Supplementary References:**

The following references support the definitions of clinical conditions and the rationale for covariate selection described in the Methods section.

S1. American Diabetes Association Professional Practice Committee. 2. Classification and diagnosis of diabetes: Standards of Medical Care in diabetes-2022. Diabetes Care 45, S17–S38 (2022).

S2. Kario, K. Key points of the 2019 Japanese Society of Hypertension guidelines for the management of hypertension. Korean Circ. J. 49, 1123–1135 (2019).

S3. Okamura, T. et al. Japan Atherosclerosis Society (JAS) guidelines for prevention of atherosclerotic cardiovascular diseases 2022. J. Atheroscler. Thromb. 31, 641–853 (2024).

S4. Yau, J. W. Y. et al. Global prevalence and major risk factors of diabetic retinopathy. Diabetes Care 35, 556–564 (2012).

S5. Tuttle, K. R. et al. Diabetic kidney disease: a report from an ADA Consensus Conference. Diabetes Care 37, 2864–2883 (2014).

S6. Pop-Busui, R. et al. Diabetic neuropathy: A position statement by the American Diabetes Association. Diabetes Care 40, 136–154 (2017).

S7. Chen, L.-K. et al. Asian Working Group for Sarcopenia: 2019 consensus update on sarcopenia diagnosis and treatment. J. Am. Med. Dir. Assoc. 21, 300–307.e2 (2020).

S8. Srikanthan, P., Horwich, T. B. & Tseng, C. H. Relation of muscle mass and fat mass to cardiovascular disease mortality. Am. J. Cardiol. 117, 1355–1360 (2016).

S9. Kim, T. N. & Choi, K. M. The implications of sarcopenia and sarcopenic obesity on cardiometabolic disease. J. Cell. Biochem. 116, 1171–1178 (2015).

S10. Park, S. W. et al. Decreased muscle strength and quality in older adults with type 2 diabetes: the Health, Aging, and Body Composition Study. Diabetes 55, 1813–1818 (2006).

S11. Mori, H. et al. High prevalence and clinical impact of dynapenia and sarcopenia in Japanese patients with type 1 and type 2 diabetes. J. Diabetes Investig. 12, 1050–1059 (2021).

S12. Pan, A., Wang, Y., Talaei, M. & Hu, F. B. Relation of smoking with total mortality and cardiovascular events among patients with diabetes mellitus: a meta-analysis and systematic review. Circulation 132, 1795–1804 (2015).

S13. Piano, M. R. Alcohol’s effects on the cardiovascular system. Alcohol Res. 38, 219–241 (2017).

S14. Steffl, M. et al. Relationship between sarcopenia and physical activity in older people: a systematic review and meta-analysis. Clin. Interv. Aging 12, 835–845 (2017).

S15. Virani, S. S. et al. Heart disease and stroke statistics—2021 update: a report from the American Heart Association. Circulation 143, e254–e743 (2021).

S16. Zhang, L. et al. Body composition and incident heart failure in older adults: results from two prospective cohorts. J. Am. Heart Assoc. 11, e023707 (2022).

S17. Cui, F. et al. Association of sarcopenia with all-cause and cause-specific mortality in cancer patients. BMC Cancer 25, 919 (2025).

S18. Alegre-Díaz, J. et al. Diabetes and cause-specific mortality in Mexico City. N. Engl. J. Med. 375, 1961–1971 (2016).

S19. Chronic Kidney Disease Prognosis Consortium. Association of estimated glomerular filtration rate and albuminuria with all-cause and cardiovascular mortality. Lancet 375, 2073–2081 (2010).
