# Supplementary material for: Age‐Stratified Associations of Sarcopenic Obesity With Mortality in Type 2 Diabetes
Source: J Cachexia Sarcopenia Muscle. 2026 Jan 28;17(1):e70211. doi: 10.1002/jcsm.70211 (PMC12848597; doi:10.1002/jcsm.70211)
Supplement: Supplementary file 2 — Table S1: Baseline characteristics of the participants in individuals aged 40–75 years. Table S2: Baseline characteristics of the participants in individuals aged over 75 years. Table S3: The association between sarcopenic obesity and all‐cause mortality after multivariable adjustment. Table S4: Firth's penalized Cox regression for the association between sarcopenic obesity and all‐cause Mortality Table S5: Distribution of causes of death according to sarcopenic obesity group. [file JCSM-17-e70211-s002.docx]

**Table S1: Baseline characteristics of the participants in individuals aged 40–75 years**

|  | All  (N=590) | Neither sarcopenia or obesity(N=318) | Obesity alone(N=246) | Sarcopenia alone(N=14) | Sarcopenic obesity(N=12) |
| --- | --- | --- | --- | --- | --- |
| Gender(male/female), n | 340/250 | 189/129 | 135/111 | 10/4 | 6/6 |
| Age, yeas | 64.3 (8.4) | 66.2 (7.4) | 61.4 (8.9) | 71.0 (3.4) | 68.0 (8.7) |
| HGS, kg | 29.5 (9.3) | 28.9 (9.0) | 31.2 (9.4) | 20.4 (6.6) | 19.4 (4.5) |
| ALM, kg | 18.9 (4.3) | 18.0 (4.0) | 20.4 (4.3) | 14.7 (3.6) | 16.8 (2.7) |
| BMI, kg/m^2 | 24.9 (4.6) | 21.9 (2.2) | 28.6 (4.1) | 22.2 (1.9) | 29.9 (3.3) |
| VFA, cm^2 | 98.2 (46.8) | 72.2 (25.4) | 129.8 (46.2) | 78.0 (34.5) | 165.9 (49.1) |
| BFP, % | 30.2(9.0) | 25.4 (7.0) | 35.9 (7.5) | 29.8 (4.4) | 43.2 (5.9) |
| SBP, mmHg | 132.9 (18.7) | 131.4 (19.9) | 134.7 (17.4) | 126.2 (14.8) | 141.7 (8.9) |
| DBP, mmHg | 77.1 (11.7) | 75.1 (11.6) | 80.1 (11.2) | 71.1 (13.6) | 76.8 (8.5) |
| Glucose, mg/dL | 152.0 (49.6) | 151.1 (49.1) | 151.7 (49.5) | 159.8 (51.8) | 171.7 (61.5) |
| Hemoglobin A1c, % | 7.5 (1.3) | 7.5 (1.2) | 7.5 (1.3) | 7.3 (1.2) | 8.0 (2.3) |
| LDL-C, mg/dL | 110.8 (30.2) | 111.5 (31.5) | 110.4 (28.6) | 103.3 (28.3) | 109.8 (33.4) |
| HDL-C, mg/dL | 58.6 (17.3) | 61.8 (18.0) | 54.8 (15.6) | 55.9 (20.2) | 54.7 (8.9) |
| TG, mg/dL | 144.6 (93.3) | 125.3 (83.0) | 170.6 (102.2) | 109.2 (52.1) | 157.8 (61.1) |
| eGFR, mL/min/1.73m^2 | 72.6 (21.6) | 72.6 (21.0) | 73.2 (21.3) | 66.0 (22.3) | 65.3 (39.1) |
| Duration, years | 13.7 (10.1) | 14.8 (10.0) | 11.7 (9.1) | 23.1 (16.4) | 16.5 (11.7) |
| Retinopathy(N=487), n | 119 (24.4) | 63 (24.0) | 49 (24.3) | 5 (38.5) | 2 (20.0) |
| Nephropathy(N=500), n | 54 (10.8) | 26 (9.8) | 21 (9.9) | 3 (23.1) | 4 (40.0) |
| Neuropathy(N=485), n | 131 (27.0) | 70 (26.7) | 53 (26.2) | 5 (45.5) | 3 (30.0) |
| CVD, n | 103 (17.5) | 48 (15.1) | 47 (19.1) | 3 (21.4) | 5 (41.7) |
| Death, n | 19 (3.2) | 11 (3.5) | 4 (1.6) | 1 (7.1) | 3 (25.0) |
| Exercise, n | 238 (40.3) | 138 (43.4) | 91 (37.0) | 5 (35.7) | 4 (33.3) |
| Alcohol habit, n | 299 (50.7) | 174 (54.7) | 112 (45.5) | 7 (50.0) | 6 (50.0) |
| Smoking status, n | 173 (29.3) | 90 (28.3) | 77 (31.3) | 5 (35.7) | 1 (8.3) |
| Hypertension, n | 319 (54.1) | 150 (47.2) | 148 (60.2) | 12 (85.7) | 9 (75.0) |
| Hyperlipidemia, n | 326 (55.3) | 164 (51.6) | 148 (60.2) | 7 (50.0) | 7 (58.3) |
| Cancer, n | 85(14.4) | 43(13.5) | 35(14.2) | 3(21.4) | 4(33.3) |

Data was expressed as mean (standard deviation) or number (%).
Participants were classified into four groups: No sarcopenic no obesity, No sarcopenic obesity, Sarcopenic no obesity, and Sarcopenic obesity.

The following abbreviations are used: ALM; appendicular lean mass, BFP; body fat percentage, BMI; body mass index, CVD; cardiovascular disease, DBP; diastolic blood pressure, eGFR; estimated glomerular filtration rate, HGS; handgrip strength, HDL-C; high-density lipoprotein cholesterol, LDL-C; low-density lipoprotein cholesterol, SBP; systolic blood pressure, TG; triglycerides, and VFA; visceral fat area.

**Table S2: Baseline characteristics of the participants in individuals aged over 75 years**

|  | All  (N=209) | Neither sarcopenia or obesity(N=131) | Obesity alone(N=30) | Sarcopenia alone(N=36) | Sarcopenic obesity(N=12) |
| --- | --- | --- | --- | --- | --- |
| Gender(male/female), n | 134//75 | 189/129 | 135/111 | 10/4 | 6/6 |
| Age, yeas | 80.7 (4.0) | 80.6 (4.0) | 79.0 (2.8) | 82.1 (4.1) | 81.7 (4.3) |
| HGS, kg | 24.7 (7.6) | 25.7 (7.5) | 28.3 (7.6) | 20.7 (5.9) | 17.2 (3.4) |
| ALM, kg | 16.6 (3.6) | 17.0 (3.5) | 18.7 (3.7) | 14.3 (3.0) | 14.2 (2.8) |
| BMI, kg/m^2 | 22.9 (3.5) | 21.6 (2.1) | 27.7 (2.5) | 21.8 (1.9) | 29.7 (4.2) |
| VFA, cm^2 | 90.2 (37.5) | 74.6 (25.1) | 127.4 (28.3) | 94.9 (33.5) | 154.3 (48.0) |
| BFP, % | 30.2 (8.2) | 26.6 (6.6) | 37.4 (4.2) | 32.0 (6.2) | 45.5 (6.1) |
| SBP, mmHg | 134.7 (16.7) | 132.5 (16.1) | 139.3 (15.6) | 136.9 (15.8) | 139.4 (25.0) |
| DBP, mmHg | 69.4 (12.2) | 68.6 (11.2) | 73.9 (14.1) | 66.9 (11.7) | 73.8 (15.9) |
| Glucose, mg/dL | 162.1 (57.0) | 152.9 (41.7) | 165.6 (55.3) | 199.4 (86.2) | 139.4 (50.8) |
| Hemoglobin A1c, % | 7.6 (1.3) | 7.6 (1.4) | 7.8 (1.4) | 7.7 (1.0) | 7.1 (0.9) |
| LDL-C, mg/dL | 100.0 (26.4) | 97.6 (28.0) | 108.0 (23.3) | 102.7 (23.5) | 97.9 (21.4) |
| HDL-C, mg/dL | 56.9 (15.3) | 58.5 (15.9) | 54.7 (17.8) | 53.4 (11.5) | 54.5 (12.0) |
| TG, mg/dL | 129.4 (75.2) | 126.1 (83.8) | 140.1 (57.0) | 126.4 (42.8) | 150.6 (93.0) |
| eGFR, mL/min/1.73m^2 | 59.6 (20.3) | 60.4 (20.4) | 60.4 (15.7) | 58.1 (23.9) | 53.7 (17.6) |
| Duration, years | 20.6 (12.2) | 22.6 (12.3) | 14.0 (9.3) | 20.8 (12.7) | 14.4 (8.0) |
| Retinopathy(N=170), n | 52 (30.6) | 39 (37.1) | 4 (16.0) | 8 (28.6) | 1 (8.3) |
| Nephropathy(N=174), n | 37 (21.3) | 21 (19.6) | 4 (15.4) | 7 (24.1) | 5 (41.7) |
| Neuropathy(N=166), n | 69 (41.6) | 40 (38.8) | 9 (36.0) | 16 (59.3) | 4 (36.4) |
| CVD, n | 37 (17.7) | 26 (19.8) | 3 (10.0) | 5 (13.9) | 3 (25.0) |
| Death, n | 22 (10.5) | 13 (9.9) | 0 (0.0) | 7 (19.4) | 2 (16.7) |
| Exercise, n | 77 (36.8) | 56 (42.7) | 6 (20.0) | 11 (30.6) | 4 (33.3) |
| Alcohol habit, n | 96 (45.9) | 63 (48.1) | 15 (50.0) | 11 (30.6) | 7 (58.3) |
| Smoking status, n | 76 (36.4) | 48 (36.6) | 8 (26.7) | 17 (47.2) | 3 (25.0) |
| Hypertension, n | 142 (67.9) | 90 (68.7) | 20 (66.7) | 21 (58.3) | 11 (91.7) |
| Hyperlipidemia, n | 130 (62.2) | 87 (66.4) | 17 (56.7) | 21 (58.3) | 5 (41.7) |
| Cancer, n | 36(17.2) | 21(16.0) | 7(23.3) | 6(16.7) | 2(16.7) |

Data was expressed as mean (standard deviation) or number (%).
Participants were classified into four groups: No sarcopenic no obesity, No sarcopenic obesity, Sarcopenic no obesity, and Sarcopenic obesity.

The following abbreviations are used: ALM; appendicular lean mass, BFP; body fat percentage, BMI; body mass index, CVD; cardiovascular disease, DBP; diastolic blood pressure, eGFR; estimated glomerular filtration rate, HGS; handgrip strength, HDL-C; high-density lipoprotein cholesterol, LDL-C; low-density lipoprotein cholesterol, SBP; systolic blood pressure, TG; triglycerides, and VFA; visceral fat area.

**Table S3. The Association between Sarcopenic Obesity and All-Cause Mortality after Multivariable Adjustment**

| **Group** | **HR (95%CI)** | ***p*** |
| --- | --- | --- |
| **Neither sarcopenia or obesity** | **Ref** |  |
| **Obesity alone** | **0.53 (0.18–1.56)** | **0.25** |
| **Sarcopenia alone** | **2.46(1.04–5.77)** | **0.04** |
| **Sarcopenic obesity** | **2.91 (1.02–8.31)** | **0.046** |

**Hazard ratios for all-cause mortality from a sensitivity analysis excluding malignancy and cardiovascular disease from the adjustment set in the primary Cox model. Estimates for the obesity-only, sarcopenia-only, and sarcopenic obesity groups were obtained from models adjusted for age, sex, diabetes duration, hemoglobin A1c, exercise habits, alcohol consumption, smoking status, hypertension, and hyperlipidemia, and were comparable in magnitude to those from the original fully adjusted model.**

**Abbreviations: Ref, reference group; CI, confidence interval; HR, hazard ratio.**

**Table S4. Firth’s Penalized Cox Regression for the Association between Sarcopenic Obesity and All-Cause Mortality**

|  | **All** | | **40-75 years** | | **> 75 years** | |
| --- | --- | --- | --- | --- | --- | --- |
| **Group** | **HR (95%CI)** | ***p*** | **HR (95%CI)** | ***p*** | **HR (95%CI)** | ***p*** |
| **Neither sarcopenia or obesity** | **Ref** |  | **Ref** |  | **Ref** |  |
| **Obesity alone** | **0.58  (0.18–1.50)** | **0.28** | **0.85 (0.25–2.41)** | **0.78** | **0.20  (0.002–1.60)** | **0.16** |
| **Sarcopenia alone** | **2.41  (0.99–5.45)** | **0.054** | **3.98 (0.39–20.7)** | **0.20** | **2.56 (0.89–6.98)** | **0.08** |
| **Sarcopenic obesity** | **3.09  (1.02–8.01)** | **0.046** | **7.88  (1.79–28.4)** | **0.009** | **1.61  (0.28–6.58)** | **0.55** |

**Firth’s penalized Cox proportional hazards regression models were used to evaluate the associations between sarcopenic obesity categories and all-cause mortality in the overall cohort and in age-stratified subgroups (40–75 and >75 years). All models correspond to the fully adjusted analysis (Model 3), which was adjusted for age, sex, diabetes duration, hemoglobin A1c, exercise habits, alcohol consumption, smoking status, hypertension, hyperlipidemia, cardiovascular disease, and cancer.**

**Abbreviations: Ref, reference group; CI, confidence interval; HR, hazard ratio.**

**Table S5: Distribution of Causes of Death According to Sarcopenic Obesity Group**

|  | CVD | Cancer | Other |
| --- | --- | --- | --- |
| All |  |  |  |
| Neither sarcopenia or obesity | 5 | 7 | 12 |
| Obesity alone | 0 | 2 | 2 |
| Sarcopenia alone | 2 | 3 | 3 |
| Sarcopenic obesity | 1 | 3 | 1 |
| Age 40–75 years (N=590) |  |  |  |
| Neither sarcopenia or obesity | 1 | 5 | 5 |
| Obesity alone | 0 | 2 | 2 |
| Sarcopenia alone | 0 | 1 | 0 |
| Sarcopenic obesity | 1 | 1 | 1 |
| Age >75 years (N=209) |  |  |  |
| Neither sarcopenia or obesity | 4 | 2 | 7 |
| Obesity alone | 0 | 0 | 0 |
| Sarcopenia alone | 2 | 2 | 3 |
| Sarcopenic obesity | 0 | 2 | 0 |

The numbers represent deaths from CVD, cancer, and other causes, stratified by sarcopenic obesity groups.

The following abbreviations are used: cardiovascular disease
