# Supplementary figures and images for: Age‐Stratified Associations of Sarcopenic Obesity With Mortality in Type 2 Diabetes
Source: J Cachexia Sarcopenia Muscle. 2026 Jan 28;17(1):e70211. doi: 10.1002/jcsm.70211 (PMC12848597; doi:10.1002/jcsm.70211)

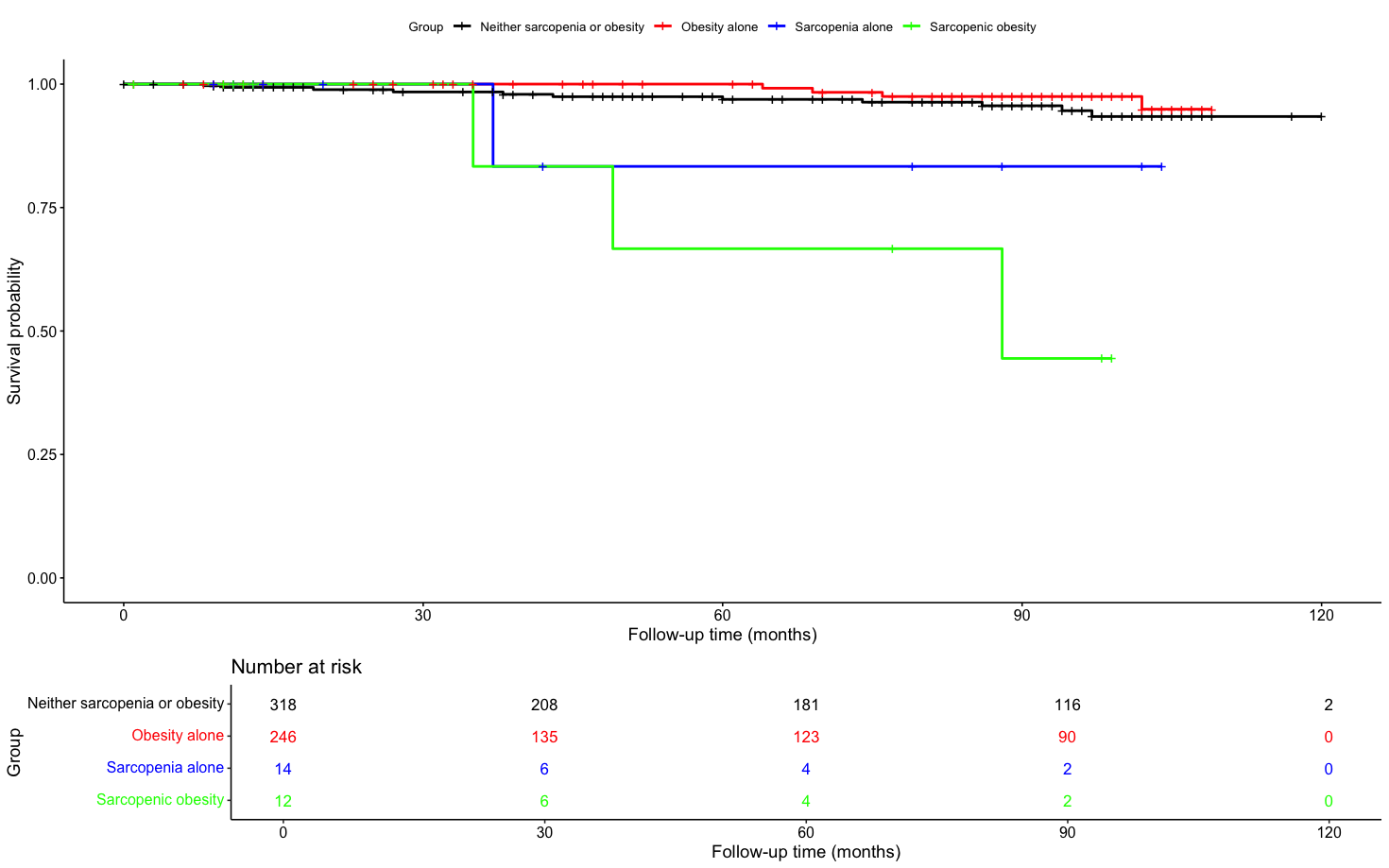

Supplement: Supplementary file 3 — Figure S1: Kaplan–Meier survival curves for all‐cause mortality stratified by sarcopenic and obese status in individuals aged 40–75 years. Cox proportional hazards regression (Model 3, fully adjusted) showed: reference = neither sarcopenia or obesity; obesity alone HR 0.57 (95% CI 0.17–1.83, p = 0.34); sarcopenia alone HR 4.41 (0.47–41.7, p = 0.20); sarcopenic obesity HR 13.1 (2.93–58.4, p < 0.001). Total number of events: 19 (neither sarcopenia or obesity = 11, obesity alone = 4, sarcopenia alone = 1, sarcopenic obesity = 3). [file JCSM-17-e70211-s004.tiff]

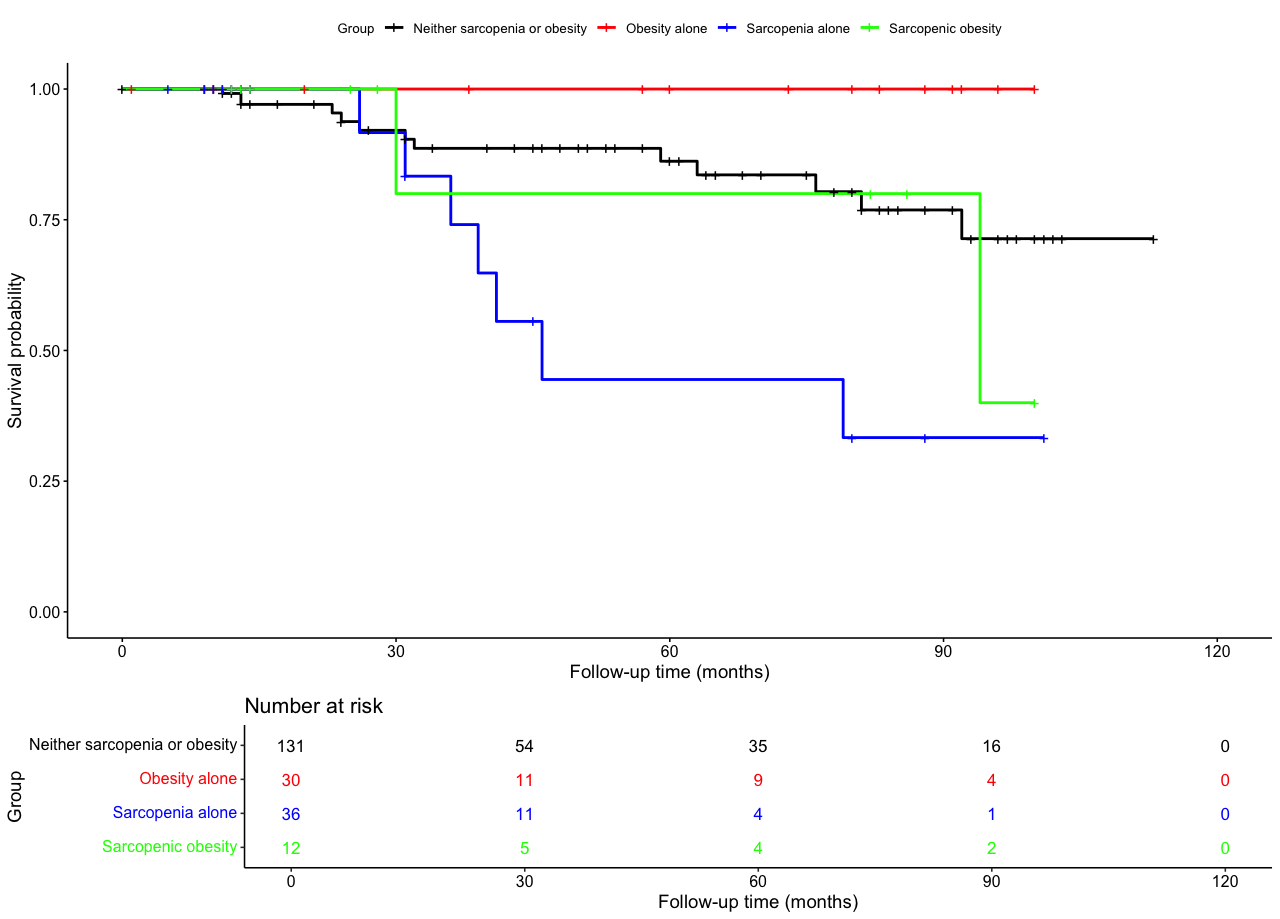

Supplement: Supplementary file 4 — Figure S2: Kaplan–Meier survival curves for all‐cause mortality stratified by sarcopenic and obese status in individuals aged over 75 years. Cox proportional hazards regression (Model 3, fully adjusted) showed: reference = neither sarcopenia or obesity; obesity alone HR 0.00 (95% CI 0.00–Inf, p = 0.99); sarcopenia alone HR 3.21 (1.07–8.33, p = 0.04); sarcopenic obesity HR 1.41 (0.34–8.81, p = 0.51). Total number of events: 22 (neither sarcopenia or obesity = 13, obesity alone = 0, sarcopenia alone = 7, sarcopenic obesity = 2). [file JCSM-17-e70211-s001.tiff]
